# Oxidative stress and antioxidant treatment in patients with peripheral artery disease

**DOI:** 10.14814/phy2.13650

**Published:** 2018-04-02

**Authors:** Panagiotis Koutakis, Ahmed Ismaeel, Patrick Farmer, Seth Purcell, Robert S. Smith, Jack L. Eidson, William T. Bohannon

**Affiliations:** ^1^ Department of Health Human Performance and Recreation Baylor University Waco Texas; ^2^ Department of Chemistry and Biochemistry Baylor University Waco Texas; ^3^ Department of Surgery Baylor Scott and White Medical Center Temple Texas

**Keywords:** Claudication, hydrogen sulfide, nitric oxide, nuclear factor (erythroid‐derived 2)‐like 2

## Abstract

Peripheral artery disease is an atherosclerotic disease of arterial vessels that mostly affects arteries of lower extremities. Effort induced cycles of ischemia and reperfusion lead to increased reactive oxygen species production by mitochondria. Therefore, the pathophysiology of peripheral artery disease is a consequence of metabolic myopathy, and oxidative stress is the putative major operating mechanism behind the structural and metabolic changes that occur in muscle. In this review, we discuss the evidence for oxidative damage in peripheral artery disease and discuss management strategies related to antioxidant supplementation. We also highlight the major pathways governing oxidative stress in the disease and discuss their implications in disease progression. Potential therapeutic targets and diagnostic methods related to these mechanisms are explored, with an emphasis on the Nrf2 pathway.

## Introduction

Peripheral artery disease (PAD) is a condition characterized by a narrowing of arteries other than those that supply the brain or the heart, most commonly affecting arteries of lower extremities (Hiatt et al. 2008). The most common underlying mechanism for PAD is atherosclerosis, and accordingly, the main risk factors for the disease are cigarette smoking, high blood pressure, diabetes, and hypercholesterolemia (Selvin and Erlinger 2004). PAD may be asymptomatic or symptomatic, and the spectrum of symptoms is classified according to the Fontaine classification (Norgren et al. 2007). Stage I is considered asymptomatic, and patients presenting with intermittent claudication (walking‐induced leg muscle pain relieved by rest) are classified in Fontaine Stage 2. In the later stages of PAD, patients exhibit foot pain at rest (Fontaine Stage 3), and/or ulcers and gangrene (Fontaine Stage 4) (Norgren et al. 2007).

Claudication and critical limb ischemia (CLI) have traditionally been thought of as consequences of reduced blood flow. However, in recent years there has been growing evidence that PAD pathophysiology is also a consequence of a compromised metabolic myopathy that affects the muscles subject to ischemia and reperfusion (Brass 1996; Brass and Hiatt 2000). Metabolically dysfunctional mitochondria of PAD patients are associated with bioenergetic decline, insufficient adenosine triphosphate (ATP) production, structural abnormalities, defects in the electron transport chain (ETC), and a decline in muscle function (Makris et al. 2007; Pipinos et al. 2008a,b). The dysfunctionality of PAD mitochondria is also associated with increased oxidative stress (Makris et al. 2007). In fact, tissue injury appears to be induced by an increased production of reactive oxygen species (ROS) in the compromised mitochondria of the affected muscles within the lower extremities (Kemp 2004).

The purpose of this review is to present the current evidence for the role of oxidative stress in the pathogenesis of PAD and discuss the value of antioxidant therapy and exercise training on the prognosis of PAD. We also discuss potential mechanisms and the major pathways of oxidative stress underlying PAD progression, including the role of the nuclear factor (erythroid‐derived 2)‐like 2 (Nrf2) master antioxidant pathway as well as activation of the receptor for advanced glycation end products (RAGE). Finally, we discuss the endogenous gasotransmitter, hydrogen sulfide (H_2_S), and its potential therapeutic role related to antioxidant, angiogenic, and vasorelaxant effects.

### Evidence for oxidative damage

Ischemia/reperfusion injury (IRI), which refers to the response that occurs in tissues that are deprived of blood flow and oxygen when circulation is restored, is a main feature of PAD. Patients with PAD experience ischemia during activity and muscle reperfusion during rest, and the restored blood flow and reoxygenation of ischemic areas is accompanied by an increase in ROS formation (Braunwald and Kloner 1985). The mitochondria generate a majority of cellular ROS (Wallace 2000), and the two major sites of ROS production are ETC complexes I and III (McLennan and Esposti 2000). Furthermore, damage to these complexes exponentially enhances the production of ROS (Moghaddas et al. 2003). Spectrophotometric and respirometric evaluations of complexes I through IV have demonstrated substantial declines in the activities of the ETC complexes I, III, and IV in PAD muscle compared to controls (Pipinos et al. 2006), which may explain the increased production of ROS. The increased ROS production may further cause cumulative mitochondrial DNA (mtDNA) damage, and progressive ETC dysfunction. An interesting point to note is that the only ETC complex that is unaffected in PAD skeletal muscle, complex II, is also the only complex encoded entirely by nuclear DNA, while all the other ETC complexes contain subunits partially encoded by mtDNA genes (Tzagoloff and Myers 1986; Tuppen et al. 2010). Therefore, damage to mtDNA could be responsible for the complex deficiencies found, as mtDNA may be exposed to high ROS levels due to its proximity to the ETC (Cooper et al. 1992). Thus, mutated mtDNA genes produce altered ETC complexes, increased ROS generation, and therefore continued injury to the mtDNA (Makris et al. 2007; Pipinos et al. 2008a).

Some of the preliminary evidence for PAD causing increased oxidative stress comes from animal models. In mice, acute ligation and excision models are used to study different severities of ischemia. For example, ligation of the femoral artery results in only mild hind limb ischemia (HLI), since blood flow may persist via collateral vessels. However, ligation and excision of the complete femoral artery and its side branches will result in severe HLI (Hellingman et al. 2010). In a mouse model of HLI investigating the effects of arterial occlusion on oxidative stress, myofibers of ischemic muscle exhibited brighter fluorescence labeling for 4‐hydroxynonenal (HNE)‐protein adducts, an end‐product of lipid peroxidation, compared with control muscle, indicating greater oxidative stress. In addition, HNE adducts measured by RPPA were two times more abundant in homogenates of ischemic muscle compared with control muscle, and quantification of carbonyl groups, which indicate protein oxidation, showed significantly increased carbonyls in ischemic muscle compared with control (Pipinos et al. 2008c). Therefore, even in the absence of comorbid conditions, occlusion of the artery was able to cause oxidative damage.

Data from humans have also established oxidative damage to myofibers as a possible cause of PAD myopathy. Oxidative damage clearly occurs in ischemic muscle (Pipinos et al. 2008a; Dopheide et al. 2013; Krishna et al. 2015). Furthermore, myofibers of PAD patients have been shown to exhibit a wide range of carbonyl and HNE damage and a greater burden of oxidative damage compared to myofibers of control patients when assessed using quantitative fluorescence microscopy (Weiss et al. 2013). After controlling for coronary artery disease and hypertension, carbonyl groups were increased 30%, and HNE adducts were increased 40% in myofibers of PAD patients compared to controls. Additionally, the extent of oxidative damage in the myofibers of PAD patients is associated with the patient's stage of disease and with a significant decrease in myofiber cross‐sectional area (Weiss et al. 2013). The same group later showed that the oxidative damage associated with PAD is myofiber‐type selective (Koutakis et al. 2014). Type I fibers are characterized by higher mitochondrial content and more oxidative energy metabolism, Type II fibers have a greater dependence on glycolysis, and Type I/II fibers are intermediate in mitochondrial content and energy metabolism (Schiaffino and Reggiani 2011). Type II and Type I/II myofibers were shown to exhibit greater oxidative damage compared to Type I myofibers in PAD muscle, and the variability among myofibers suggested the possibility of selective damage to Type II fibers. Type II fibers also demonstrated the highest increase in carbonyl content among the different myofiber types, compared to controls. A frequency analysis of Type I, Type II, and Type I/II myofibers demonstrated a reduction in the ratio of Type II to Type I myofibers in PAD, providing further support for selective damage to Type II myofibers. These results are consistent with other studies showing increased sensitivity of Type II fibers to oxidative damage (Anderson and Neufer 2006; Stefanyk et al. 2010) as well as a preferential reduction in Type II muscle fiber size and frequency in disease (Wang and Pessin 2013). These findings may explain possible mechanisms behind the chronic and progressive nature of PAD, as accumulating oxidative damage in the ischemic limb may cause the patient to transition from fast to slow twitch fibers. Oxidative stress markers have been strongly correlated with the severity of PAD and inversely associated with absolute and pain‐free walking distance (Dopheide et al. 2013). Preliminary data from a survival analysis of a population of PAD patients also identify oxidative myofiber damage, measured as protein carbonyl groups of the gastrocnemius, as a predictor of mortality rate independent of other covariates and measures, including the ankle‐brachial index (ABI), a test used to diagnose PAD by measuring the ratio of the blood pressure of the ankle to the blood pressure of the upper arm (Koutakis et al. 2016). Taken together, these data further raise the importance of oxidative stress in the pathogenesis and mortality associated with PAD and suggest that myofiber degeneration may be a consequence of the accumulation of oxidative damage within the fibers.

## Management of Oxidative Stress

### Antioxidants

Furthermore evidence for the important role that oxidative stress plays in PAD comes from studies evaluating antioxidants and antioxidant defense systems. Of the primary enzymatic antioxidant defenses, manganese superoxide dismutase (MnSOD) is the first line of ROS defense in mitochondria, and MnSOD has been shown to be deficient in PAD muscle (Pipinos et al. 2006). In addition, plasma glutathione peroxidase (GPX) activity, selenium, an essential cofactor of GPX, and glutathione transferase, an enzyme that helps restore GPX, are significantly decreased in PAD patients compared to controls (Pessah‐Rasmussen et al. 1993; Edwards et al. 1994). Specific antioxidants are also altered in patients with PAD. For example, vitamin C is a sacrificial antioxidant that is consumed during oxidative stress, and significant oxidative stress may be inferred from reduced concentrations of vitamin C (Frei 1994). Indeed, lower vitamin C concentrations were observed in intermittent claudication patients. Interestingly, this was in association with higher levels of the inflammatory marker C‐reactive protein (CRP) as well as PAD severity, measured by both ABI and initial claudication distance, suggesting a link between oxidative stress and functional capacity in PAD patients (Langlois et al. 2001). Similar results of reduced antioxidant content were seen with other antioxidants, including alpha‐tocopherol (Larsson and Haeger 1968), glutathione (Judge and Dodd 2003), bilirubin and albumin (Krijgsman et al. 2002), and total antioxidant capacity, or the sum of endogenous and food‐derived antioxidants (Spark et al. 2002). Taken together, a dysfunctional antioxidant defense system that is unable to protect against oxidizing species may be an important hallmark of PAD.

Given the clear evidence of oxidative stress in PAD, antioxidant supplementation may be a viable countermeasure. Early studies using high doses of vitamin E in patients with PAD led to improved exercise tolerance (Livingstone and Jones 1958; Haeger 1968; Williams et al. 1971). More recently, administration of 200 mg of vitamin E along with 500 mg of vitamin C for 4 weeks was shown to reduce exercise‐associated oxidative stress (Wijnen et al. 2001), as did an intravenous infusion of vitamin C at a dosage of 50 mg/min for 20 min (Silvestro et al. 2002). Infusion of glutathione (GSH), a major endogenous antioxidant and the primary defense against oxidants in skeletal muscle, twice a day for 5 days increased initial claudication distance by 37% compared with saline‐treated patients and improved macrocirculatory flow and post‐ischemic hyperemia (Arosio et al. 2002). While nonspecific antioxidants seem to be successful in PAD patients, direct antioxidant therapies with antioxidant concepts such as Nrf2 activators (Houghton et al. 2016) (discussed further in later sections) have not yet been investigated so far in human PAD. Future clinical studies should establish the effects of these novel strategies on oxidative stress in PAD patients.

### Exercise

Exercise training is generally believed to be highly beneficial to the prognosis in most cardiovascular diseases, via a decrease in oxidative stress and inflammation (Sallam and Laher 2016). For PAD specifically, exercise is also considered a primary treatment option (IA rating) given the evidence for improved health following exercise therapy (Norgren et al. 2007). In fact, supervised treadmill exercise is thought to have greater benefit than medications and is recommended as first‐line therapy to improve walking performance in PAD (McDermott and Polonsky 2016). Home‐based exercise training for 12 months has been shown to ameliorate inflammation, reduce ROS, and increase walking distance (Dopheide et al. 2015). Furthermore, a follow‐up study comparing supervised and nonsupervised training showed that the supervised exercise training was even more efficient in improving the inflammatory status of PAD patients and their walking distance, although both forms of exercise had beneficial effects (Dopheide et al. 2016).

In most of the successful interventions of exercise for patients with PAD, the outcome measures assessed are typically pain‐free walking time and quality of life (McDermott and Polonsky 2016). However, there is at least some evidence that exercise may not help PAD patients, or may even exacerbate the damage in their legs, especially when assessing the effects of exercise on oxidative stress and morphological changes. For example, while a 12‐week program of exercise therapy demonstrated significantly increased peak exercise performance and peak oxygen consumption, when evaluating the gastrocnemius biopsy specimens, greater skeletal muscle injury was also seen as a result of exercise (Hiatt et al. 1996). Likewise, after a single bout of exercise‐induced claudication, ROS biomarkers have been shown to increase in the plasma of PAD patients (Hickman et al. 1994; Silvestro et al. 2002). Results such as these have led some researchers to question whether exercise may actually induce metabolic, oxidative, and inflammatory stress in the already damaged and impaired PAD limb, thereby worsening the condition of the ischemic muscle (Hamburg and Balady 2011). Therefore, there is a need for studies that will establish the optimal exercise prescription for claudicating patients by both evaluating the effects of exercise on skeletal muscle histology as well as changes in performance.

## Mechanisms

### ROS as signaling molecules

While ROS are generally thought of in a cytotoxic light as they can lead to oxidative damage and cell death in high levels, ROS at low levels function as signaling molecules in redox regulation (Trachootham et al. 2008). Redox signaling may be important specifically for PAD, as the pathology of PAD encompasses ischemia reperfusion, which is understood to be a redox regulated process (de Vries et al. 2013; Görlach et al. 2015). ROS are necessary for angiogenesis mediated by vascular endothelial growth factor (VEGF), implicated in angiogenesis of ischemic tissue (Marti et al. 2000; Shibuya 2011; Talwar and Srivastava 2014). For example, when ROS are decreased either by the use of antioxidants or by inhibition of NADPH oxidase, VEGF activity, and revascularization are decreased (Abid et al. 2007). Likewise, hypoxia‐inducible factor (HIF‐1), a protein that is considered the master transcriptional regulator of cellular response to hypoxia as well as tissue regeneration (Zhang et al. 2015), requires certain levels of ROS for stabilization (Diebold et al. 2010a,b), and vascular remodeling by HIF‐1 is induced by ROS.

There is evidence that some ROS generating NADPH oxidases, such as Nox4 (a source of H_2_O_2_) function to protect vascular function following ischemia. For example, ROS derived from Nox2, an NADPH oxidase, are required for upregulation of HIF‐1, and antioxidants and Nox2 knockdown can prevent increased angiogenesis (Diebold et al. 2012). In addition, Nox4‐deficient mice show attenuated angiogenesis, and H_2_O_2_ has been shown to be an important player in promoting angiogenesis (Schröder et al. 2012; Murdoch et al. 2014). HLI of mice can also increase Nox2 expression and ROS production, and Nox2‐deficient mice show decreased ROS levels and reduced flow recovery and capillary density (Urao et al. 2008). Furthermore, following HLI surgery, mice with upregulated glutaredoxin‐1 (Glrx), an antioxidant enzyme, exhibit reduced blood flow recovery and capillary density partly due to altered VEGF signaling (Murdoch et al. 2014). Glrx overexpression also prevents HIF‐1 stabilization, and Glrx deletion increases HIF‐1 levels and the expression of angiogenic genes (Watanabe et al. 2016). Therefore, ROS may be understood to play two contrasting roles in ischemia. While some levels of ROS are required as signaling molecules to mediate important angiogenic mechanisms, excessive amounts can cause oxidative stress that results in impaired regeneration of muscle fibers and potentially limb loss.

### Nrf2

Detoxification proteins bearing antioxidant response elements (AREs) in their promoter regions are an established and critical component to cellular antioxidant defense mechanisms. One of the principal regulators of the ARE is the transcriptional factor Nrf2. Nrf2 binds to the ARE in the regulatory regions of target genes and to Kelch ECH associating protein (Keap1). Modifications in Keap1 lead to Nrf2 activation and nuclear translocation for subsequent target gene expression (Kansanen et al. 2013). Nrf2 induces transcriptional activation of a number of ARE‐binding antioxidants such as NADPH dehydrogenase, SOD, and GPX (Howden 2013). Referred to as the “master regulator” of the antioxidant response and responsible for modulating the expression of hundreds of genes, Nrf2 is considered an essential aspect in the pathogenesis of cardiovascular diseases (Hybertson et al. 2011). The dysregulation of Nrf2‐regulated genes is thought to provide a link between oxidative stress and several human diseases.

**Figure 1:**
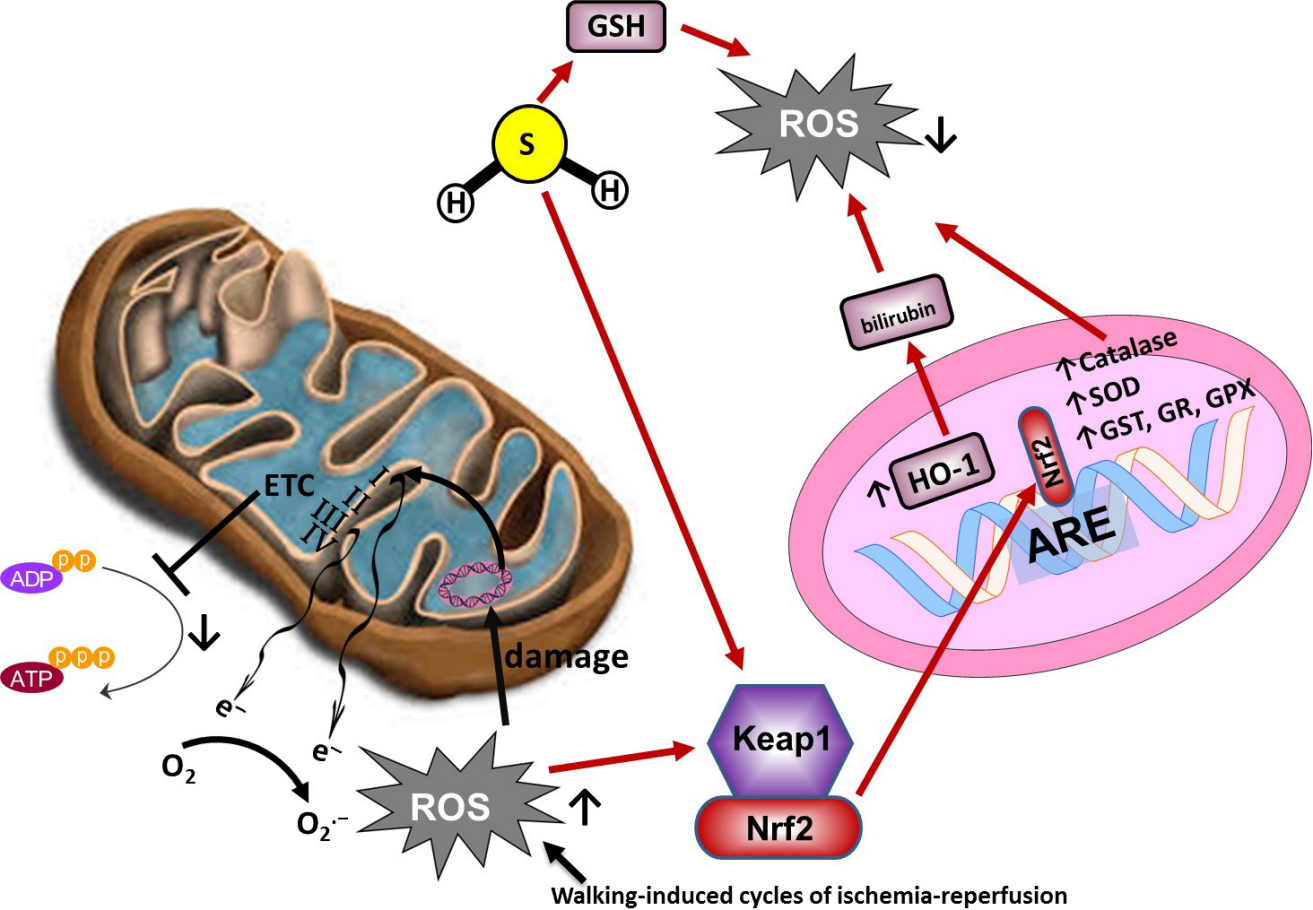
Major pathways governing oxidative stress in PAD. Effort induced cycles of ischemia and reperfusion lead to increased reactive oxygen species production by mitochondria, with the two major sites of ROS production being ETC complexes I and III. The increased ROS production may further cause cumulative mtDNA damage, and progressive ETC dysfunction. Nrf2 may be a key component in attenuating oxidative stress in ischemia‐reperfusion injury. H2S can increase localization of Nrf2, modulating the expression of many antioxidant genes, as well as activation of the HO‐1 signaling pathway.

Nrf2 has specifically been implicated as an important candidate for protection against IRI, since blood flow restoration causes a significant increase in oxidative stress that can overwhelm antioxidant defenses (Fig. 1). While there is little information about the role of Nrf2 in IRI, the evidence that exists does support Nrf2 as a key component in attenuating oxidative stress in IRI. Significant reductions in Nrf2 activation have been shown in skeletal muscle of CLI patients (Islam et al. 2015). Notably, this reduction was accompanied by significant reductions in antioxidant proteins as well as significant increases in biomarkers of oxidative stress (Islam et al. 2015). Furthermore, in a rat cardiac cell model of IRI, cells treated with the phase II antioxidant enzyme inducer D3T showed a significant reduction in ROS. These cells also demonstrated increases in Nrf2 mRNA and protein, suggesting a potential role for Nrf2 in protecting against the accumulation of ROS following IRI (Cao et al. 2006). Acute activation of Nrf2 has also been shown to have cardioprotective effects following IRI. For example, when mice were treated with H_2_S before myocardial ischemia, the H_2_S increased the localization of Nrf2 and led to significant decreases in infarct size and oxidative stress (Calvert et al. 2009). Likewise, when 4‐HNE was used to activate Nrf2 in a cardiomyocyte model, 4‐HNE improved functional recovery following IRI, and the cardioprotective effect was not observed in Nrf2‐knockout mice (Zhang et al. 2010). Despite these beneficial results, however, other studies have also shown that chronic activation of Nrf2 may be detrimental to cardiac function (Howden 2013). Therefore, while future studies should investigate Nrf2 further as a potential therapeutic target for PAD, it is important to first establish and clearly elucidate the role for Nrf2 in the pathogenesis of cardiovascular diseases.

Heme oxygenase‐1 (HO‐1) is an important antioxidant enzyme that plays a role in protection against oxidative stress (Wenzel et al. 2007). Nrf2 is a critical regulator of HO‐1, achieved by binding to the ARE, and for this reason, HO‐1 and Nrf2 are often referred to together as the Nrf2/HO‐1 system (Kim et al. 2010; Loboda et al. 2016). HO‐1 also catalyzes the oxidative degradation of heme to biliverdin IX*α*, which is later reduced to bilirubin (Nakayama et al. 2001). Bilirubin can protect against oxidation of lipids, and its antioxidant effect exceeds that of vitamin E toward lipid peroxidation (Stocker et al. 1987; Sedlak and Snyder 2004). In addition to its protective role against oxidative stress, HO‐1 also contributes to angiogenesis, and the HO‐1 signaling pathway has been implicated in modulating the risk for cardiovascular diseases (Idriss et al. 2008; Durante 2010; Fredenburgh et al. 2015). HO‐1 has also been shown to be significantly reduced in PAD patients, and has been implicated as an independent predictor of PAD (Signorelli et al. 2016). It is likely that the reduction in HO‐1 is part of a compensatory mechanism to maintain the cellular redox status, and HO‐1 may be a useful tool in determining the presence of PAD.

### AGEs

Advanced glycation end products (AGEs) are proteins or lipids formed by nonenzymatic glycation or oxidative reactions and have been shown to contribute to a number of chronic diseases by promoting cellular dysfunction via binding to cellular surface receptors (Bierhaus et al. 2005). The RAGE acts as a counter‐receptor for AGEs, and engagement of the receptor results in cellular dysfunction and tissue destruction (Bierhaus et al. 2005). The formation of AGEs is known to increase ROS formation and impair antioxidant systems, and the formation of some AGEs is induced under oxidative conditions (Nowotny et al. 2015).

**Figure 2:**
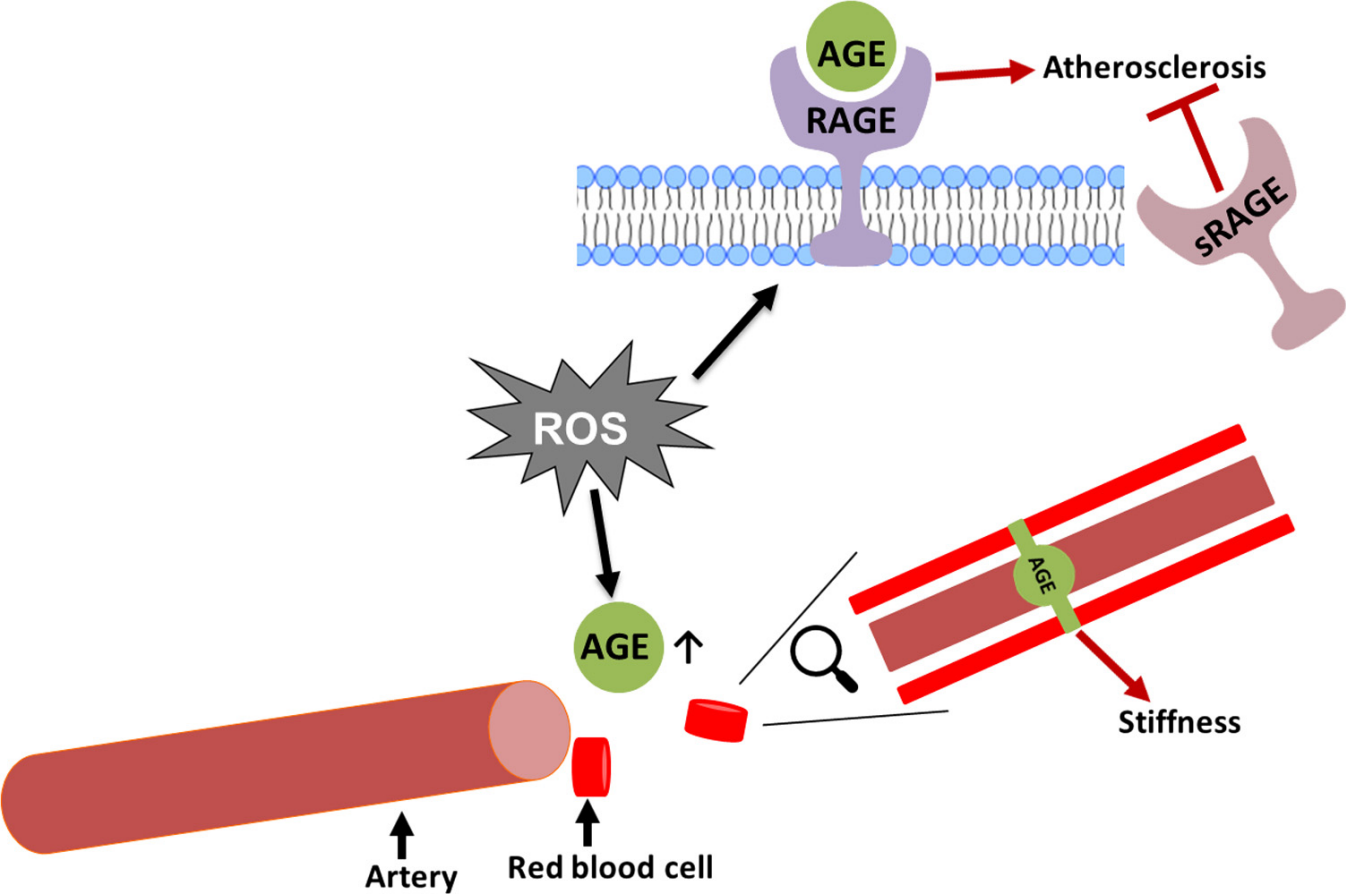
AGEs formed by oxidative reactions may promote cellular dysfunction and tissue destruction by binding to the RAGE, leading to atherosclerosis and increased arterial stiffness. Pathological effects mediated via RAGE are inhibited by the decoy receptor soluble RAGE (sRAGE).

AGEs have been implicated as a group of biomarkers important in identifying patients with increased cardiovascular risk. The formation of AGEs accompanies diabetes mellitus (DM) and is believed to be one contributing factor for the role of oxidative stress in hyperglycemia‐induced tissue injury in DM. Furthermore, patients with type 2 DM have a high risk for early and extensive development of PAD, and activation of the RAGE could be a mechanism for this accelerated PAD (de Vos et al. 2016). AGEs have been shown to be elevated in the serum of type 2 diabetic patients with PAD, with a simultaneous reduction in antioxidant status (Lapolla et al. 2007). Other studies have used skin autofluorescence (SAF) as a non‐invasive measurement of tissue AGE accumulation, and SAF has been reported to be significantly increased in PAD patients (Noordzij et al. 2012; Liu et al. 2015a; de Vos et al. 2016). Additionally, in a 5‐year prospective cohort study, higher SAF was independently associated with increased risk for all‐cause mortality and fatal or nonfatal major adverse cardiovascular events (de Vos et al. 2014), and SAF was a predictor for amputation in patients with PAD independent from the presence of DM and Fontaine stage (de Vos et al. 2015). Cilostazol, an antiplatelet, antithrombotic agent that is used in the treatment of PAD, has been shown to attenuate the severity of PAD through augmentation of the decoy receptor soluble RAGE (sRAGE) (Liu et al. 2015b). In addition to using AGEs as a biomarker, lowering of AGEs may also be a potential therapeutic target for PAD patients (Fig. 2).

### H_2_S

**Figure 3:**
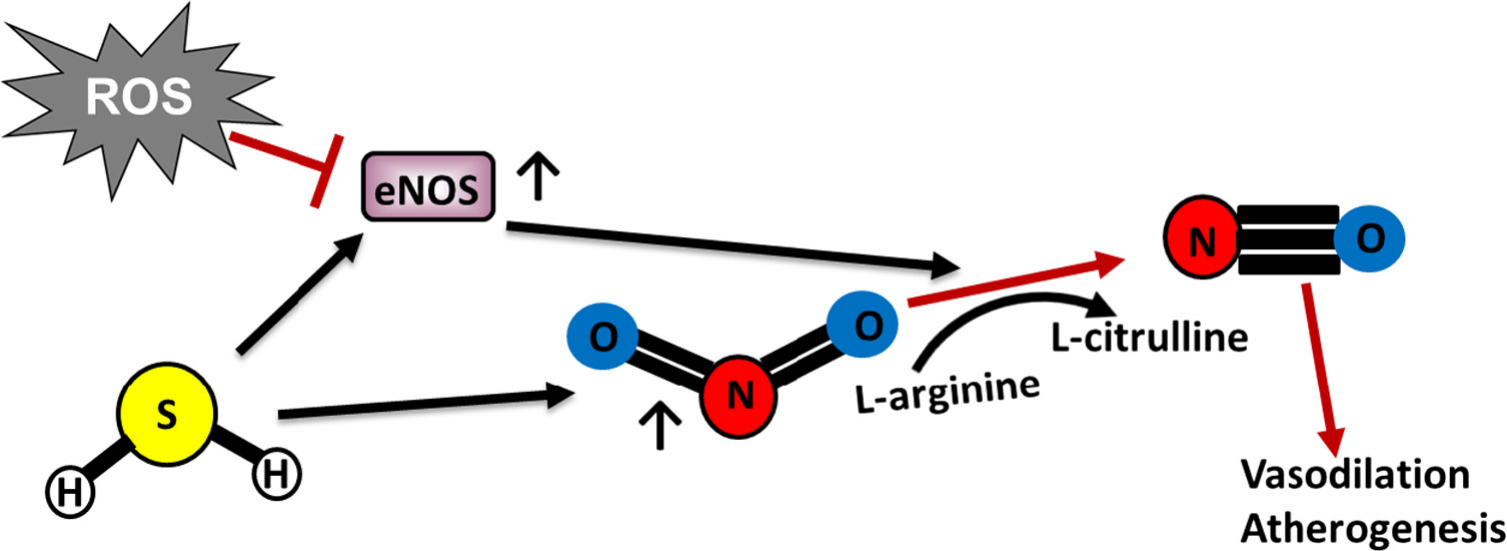
H_2_S holds promise as a therapeutic agent in PAD. Via its role in Nrf2 nuclear localization and signaling, H_2_S may prove to be an important target in combating oxidative stress accompanying the disease. Additionally, H_2_S regulates eNOS expression and function and increases nitrite reduction to NO, therefore playing a role in atherogenesis.

Hydrogen sulfide (H_2_S) has recently received much attention for its antioxidant function, as it is able to scavenge ROS, thus preventing oxidative stress (Yang et al. 2015). H_2_S has also been shown to increase GSH production, decrease NADPH oxidase activity, decrease ROS production, and protect from oxidative stress in different conditions (Kimura and Kimura 2004; Yan et al. 2006; Kimura et al. 2009; Wen et al. 2013). In addition, H_2_S plays a role in vasodilation (Wang et al. 2009), the regulation of atherogenesis (Coletta et al. 2012), endothelial nitric oxide synthase (eNOS) expression and function (Bir et al. 2012), Nrf2 nuclear localization and signaling (Calvert et al. 2009), and it increases nitrite reduction to NO (Cortese‐Krott et al. 2015) (Fig. 3). Notably, eNOS‐derived NO mediates vasorelaxation and is an important mediator of antiproliferative effects induced by proinflammatory cytokines (Quintana‐Lopez et al. 2013; Daiber et al. 2017bb). Increased oxidative stress as well as hypoxia, both features of IRI, can lead to eNOS uncoupling, and the formation of more ROS instead (Daiber et al. 2017a).

H_2_S is believed to be involved in the pathogenesis of atherosclerotic disease, including PAD, and H_2_S bioavailability is attenuated in CLI skeletal muscle (Islam et al. 2015). H_2_S has also been shown to be involved in the development and attenuation of consequences of ischemic vascular remodeling and IRI (Wang et al. 2009; Bir et al. 2012; Wang 2012). Studies have shown H_2_S to decrease oxidation of low‐density lipoprotein (LDL), uptake of oxidized LDL by macrophages, foam cell formation (Laggner et al. 2007; Zhao et al. 2011), and vascular calcification (Wu et al. 2006). Mice subjected to HLI treated with diallyl trisulfide (DATS), an organic polysulfide which liberates H_2_S, were show to have enhanced blood flow recovery and capillary density in ischemic limbs, as well as reduced oxidative stress in ischemic muscles (Hayashida et al. 2017). Addition of DATS to cultured human umbilical vein endothelium cells also decreased oxidative stress under hypoxic conditions via stimulation of Akt and eNOS (Hayashida et al. 2017). H_2_S has been implicated in hypoxia‐mediated damage by regulating functions of hypoxia‐inducible factors and acting as an important oxygen/hypoxia sensor (Yang et al. 2015). Taken together, this emerging evidence suggests that changes in H_2_S may affect several different pathophysiological responses involved in atherosclerotic vessel diseases.

Another study that measured H_2_S levels in the plasma of PAD patients showed that free‐plasma H_2_S levels in patients with PAD alone (mean 514.4 nmol/L) were significantly greater than patients without vascular disease (mean 368.5 nmol) (Peter et al. 2013). However, plasma total NO levels were significantly lower only in patients with PAD alone (mean 38.86 nmol/L) compared to subjects without vascular disease (mean 64.7 nmol/L), suggesting the H_2_S/NO ratio may be altered in PAD. Notably, H_2_S elevation may be a result of a compensatory mechanistic response to endothelial dysfunction, since H_2_S can regulate NO bioavailability (Bir et al. 2012; Coletta et al. 2012). Finally, it is important to note that elevated H_2_S levels may also play an unfavorable role in PAD, as H_2_S may interact with NO metabolites to generate novel reactive sulfur species (Filipovic et al. 2012; Giles et al. 2017), adding another layer of complexity to consider in terms of pathophysiological implications.

## Conclusions

Based on the reviewed data, oxidative stress related to ischemia reperfusion is likely a major operating mechanism of PAD myopathy. Repeated cycles of ischemia/reperfusion over time lead to structural myofiber damage and eventually severe myopathy, including a shift in myofiber type. Several measures of oxidative damage have been implicated as biomarkers and diagnostic tools for the disease. Compromised antioxidant defense systems, which are unable to respond to the elevated ROS production, seem to accompany PAD as well. While antioxidant strategies may be successful in PAD patients, studies elucidating the important role of ROS in angiogenesis and revascularization via redox signaling mechanisms emphasize caution in the use of antioxidants, or at least high‐dose administration of antioxidants.

Antioxidants targeting the Nrf2 pathway, remain an area with very little research. Nrf2 may be a novel therapeutic target in the treatment of PAD, as acute activation has been shown to be cardioprotective following ischemia reperfusion. Stabilization and greater nuclear accumulation of Nrf2 modifies gene expression and leads to greater synthesis of antioxidants and antioxidant enzymes, greater elimination of ROS, and improved angiogenesis. Notably, H_2_S holds promise as a clinically useful biomarker for PAD that may also hold therapeutic benefits. Via its role in Nrf2 nuclear localization and signaling, H_2_S may prove to be an important target in combating the increased oxidative stress that accompanies the disease. Additionally, H_2_S may be an important player in atherogenesis by modulating NO levels. The findings presented here serve as a basis for future studies to further examine H_2_S and NO levels in PAD patients to better elucidate the pathophysiological importance of the compounds and their bioavailability during the disease.

## Conflict of Interest

The authors have no conflict of interest to declare.
